# Comparative analysis of surface coating properties of five hydrophobins from *Aspergillus nidulans* and *Trichoderma reseei*

**DOI:** 10.1038/s41598-018-29749-0

**Published:** 2018-08-13

**Authors:** Lex Winandy, Felix Hilpert, Oleksandra Schlebusch, Reinhard Fischer

**Affiliations:** 1 Department of Microbiology, Institute for Applied Biosciences, Karlsruhe Institute of Technology (KIT), Karlsruhe, Germany; 2 Institute of Chemical Process Engineering, Mannheim University of Applied Sciences, Mannheim, Germany

## Abstract

Fungal hydrophobins are small amphiphilic proteins that self-assemble into monolayers on hydrophobic:hydrophilic interfaces and can be used for surface coatings. Because e.g. *Aspergillus nidulans* contains six different hydrophobins, it is likely that they have different properties and are used for different “applications” in the fungus. We established a method for recombinant production of different class hydrophobins in *Escherichia coli*. We produced DewA, DewC, DewD, DewE from *A*. *nidulans* and HFBI from *Trichoderma reesei* and compared surface coating properties of these hydrophobins. All tested proteins formed coatings on glass, strongly increasing the hydrophobicity of the surface, and showed emulsion-stabilizing properties. But whereas the typical class I hydrophobin DewA formed the most stable coating on glass, the intermediate class hydrophobins DewE and DewD were more effective in stabilization of oil:water emulsions. This work gives insights into correlations between structural characteristics of hydrophobins and their behaviour as surface binding agents. It could help with the clarification of their biological functions and lead to novel biotechnological applications.

## Introduction

Hydrophobins are small amphiphilic proteins that self-assemble into monolayers on hydrophilic and hydrophobic surfaces and change their properties^1–3^. Fungi secrete these proteins to reduce surface tension and support hyphae growth or to increase the hydrophobicity of conidiospores, aerial hyphae and fruiting bodies^2,4,5^.

Hydrophobins are cysteine rich proteins that are characterized by four intramolecular disulfide bridges^6^. Depending on their structural features, like for example the lengths of the loop between the cysteine residues, hydrophobins are divided into two classes^6^. However, several hydrophobins have been identified that do not fit into either of the two classes, like for example the protein DewD from *A*. *nidulans*^4,7,8^. Generally, class I hydrophobins form highly stable layers that can withstand detergents and high temperatures. Their formation involves conformational changes of the protein molecules upon interaction with one another and formation of amyloid fibrillar structures^9,10^. These layers on conidiospore surface are highly structured and shaped into so called rodlets, approximately 10 nm in hight. The layers formed by the class II hydrophobins are less stable and can be dissolved by ethanol, detergents or pressure^11–13^.

Various applications, that have been investigated for hydrophobins, include coating and modification of solid surfaces, emulsion and foam stabilization, increasing enzyme activity or antifouling^14–18^. However, the preparation of hydrophobins from wild type strains is complex and results mostly in milligram amounts of purified protein, due to the low natural production levels or strong binding of the proteins to the fungal cell wall^13,19^. Heterologous production of hydrophobins was first attempted in *Escherichia coli* with the *Neurospora crassa* class I hydrophobin EAS^20^. Recently a method of industrial-scale production in *E*. *coli* has been developed with modified DewA hydrophobin from *A*. *nidulans*^21^. Time-consuming and expensive downstream purification steps prior to hydrophobin usage have been reported for HGFI^22^ and CMiI1, CMiI2 and CMiI3, all produced in *P*. *pastoris*^23^. It has also been reported for two Class II hydrophobins HFB4 and HFB7 from *T*. *virens* that the production host, *E*. *coli* or *P*. *pastoris*, influences the surface binding properties of produced proteins^24^. No production method for hydrophobins has been universally established so far.

*A*. *nidulans* possesses six hydrophobins that are present on the conidiospore surface^4^. Most of these hydrophobins belong to class I can potentially be used for highly stable surface functionalization. DewA contributes to the spore hydrophobicity and has been so far established as the first-choice hydrophobin from *A*. *nidulans* for biosynthetic surface modification^14,15,25,26^. Hydrophobins RodA and DewB both possess a glycosylphosphatidylinositol (GPI) anchor for immobilization on the spore surface and both contribute to the hydrophobicity of the conidiospore surface of *A*. *nidulans*^4^. Due to the anchor they are less suitable for application in soluble form. The deletion of the anchor, as shown for the DewB protein, results in almost complete loss of surface binding properties for this protein^14^. The function and surface binding properties of other hydrophobins from *A*. *nidulans* are less studied. They are all present on the spore surface, with DewD and DewE also expressed in hyphae^4^. Several of them (DewA, DewB, RodA and DewC) are induced in the presence of lignocellulose, with RodA and DewC directly contributing to *A*. *nidulans* growth on lignocellulose^14,27^. It can be assumed that they all fulfil specific functions and that their biochemical or biophysical properties vary. Likewise, in for example *Schizophyllum commune* the Sc3 hydrophobin is expressed in aerial hyphae and the Sc1 and Sc4 hydrophobins are expressed in hyphae of fruiting bodies, suggesting different cellular functions and interesting specific properties of each hydrophobin^28^.

In this study we have implemented a universal method for heterologous production and purification of soluble class I hydrophobins (DewA, DewC, DewE from *A*. *nidulans*), an unknown class hydrophobin (DewD from *A*. *nidulans*) and class II hydrophobin (HFBI from *T*. *reseii*) in *E*. *coli*. To assess the suitability of different hydrophobins for biosynthetic surface modification, we have characterized their surface binding properties and analysed the long-termed stability of the formed layers and their resistance towards temperature, UV light, ethanol and detergent. We have also tested the stabilizing effect of these proteins on water:oil emulsions to test their potential application as emulsion or foam stabilizers.

## Results

### Design of modified hydrophobins for production in *E*. *coli*

**Figure 1:**
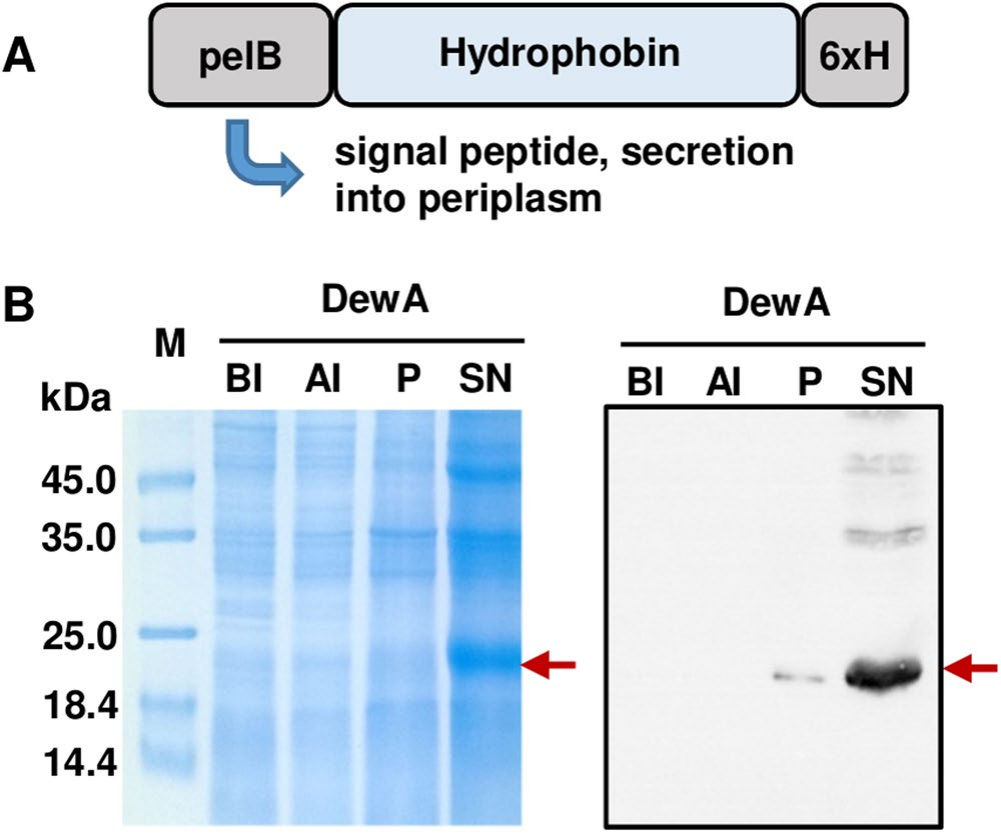
Recombinant hydrophobin production. (**A**) Design outline of the expressed hydrophobins with N-terminal pelB leader sequence and C-terminal 6xHis tag for detection. (**B**) Detection of the hydrophobin DewA during purification by Coomassie stained 15% SDS-polyacrylamide gel and protein immunoblot detection (15 sec exposure). BI - before induction, AI - after induction, P - pellet after inclusion body purification, SN –soluble protein in supernatant after purification. Arrow indicates the DewA protein monomer. Full-length gel and blot are presented in Supplementary Figure S1.

Efficient heterologous production of hydrophobins often requires modifications of the original protein sequence to ensure the correct biosynthesis and intracellular localization suitable for purification of these cysteine-rich amphiphilic proteins. We have modified the hydrophobins DewA, DewC, DewE, DewD from *A*. *nidulans* and HFBI from *T*. *reseii* by fusing them N-terminally with the pectate lyase B leader sequence *pelB*^29^ and C-terminally with a His-tag (Fig. 1A). It has been shown previously that the fusion of *pelB* to the HIV-1 protein Vpu is a suitable method for the expression of membrane-targeted proteins in *E*. *coli*, and also Class II hydrophobins^24,30^. Fused to PelB the proteins are directed to the bacterial periplasm from where they can be purified by denaturation and renaturation from inclusion bodies. Compared to another peptide modification with the YaaD fragment from *Bacillus subtilis*, introduced for hydrophobin purification in *E*. *coli*^21^, the PelB peptide is smaller and is less likely to interfere with the surface binding properties of the hydrophobins. The His-tag was added to the hydrophobins for immunodetection and potential downstream purification steps, for example if the crystallization of the protein would be eligible. The purification of recombinant hydrophobins was carried out from *E*. *coli* periplasm using alkaline pH and resulted in almost complete solubilization of the produced hydrophobins (Fig. 1B). From one liter culture following hydrophobin concentrations have been achieved in final volume of 20 ml each: 2.3 mg/ml DewA, 3.4 mg/ml DewC, 2.1 mg/ml DewD, 1.9 mg/ml DewE, 1.3 mg/ml HFBI.

### Fluorescence microscopy of hydrophobin coated glass

**Figure 2:**
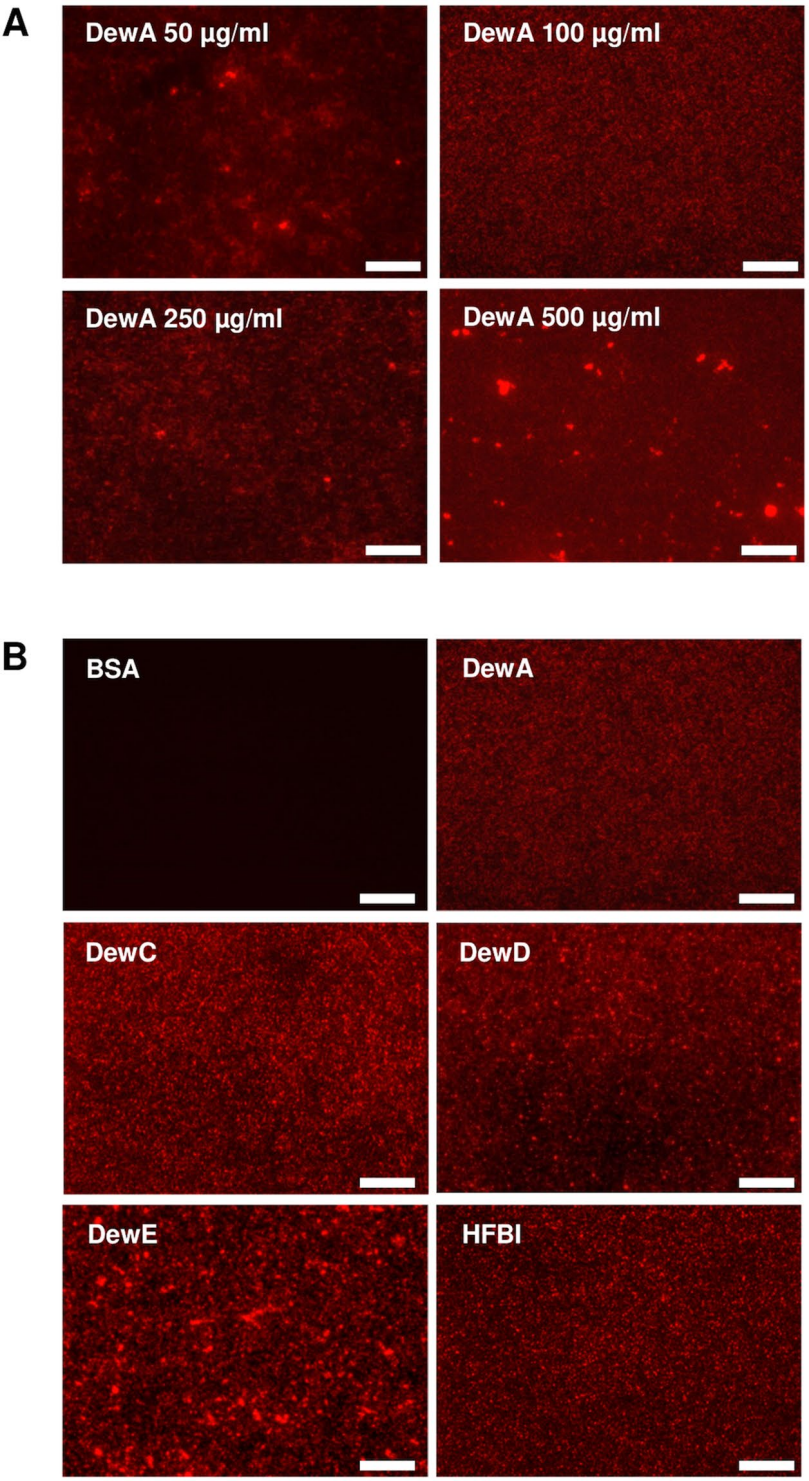
Hydrophobin coating on glass slides visualized by fluorescence microscopy. (**A**) DewA coated glass slides with different protein concentrations in the coating solution. (**B**) Glass slides coated with the 5 different hydrophobins with a protein concentration of 100 µg/ml. BSA was used as negative control. Scale bar = 20 µm.

To determine the protein concentration suitable to generate even and uniform hydrophobin coatings, clean glass slides were coated with DewA solution with different protein concentrations ranging from 50 µg/ml up to 500 µg/ml. Hydrophobin coatings were visualized by fluorescence microscopy with an α-His primary antibody and a Cy3-labelled secondary antibody. A uniform layer was achieved with protein concentrations of 100 µg/ml and 200 µg/ml (Fig. 2A). Lower concentrations led to an uneven coating, whereas higher concentrations resulted in the formation of big hydrophobin aggregates on the surface. The protein concentration of 100 µg/ml was chosen for all further surface coating experiments. The results achieved with the hydrophobins DewC, DewD, and HFBI did not differ substantially from the DewA layers at 100 µg/ml concentration (Fig. 2B). DewE coating showed, however, a more uneven and grained layer structure on glass surface. This result could not be altered by lower or higher DewE concentration in the coating solution (see Supplementary Fig. S2).

### Atomic force microscopy of coated surfaces

**Figure 3:**
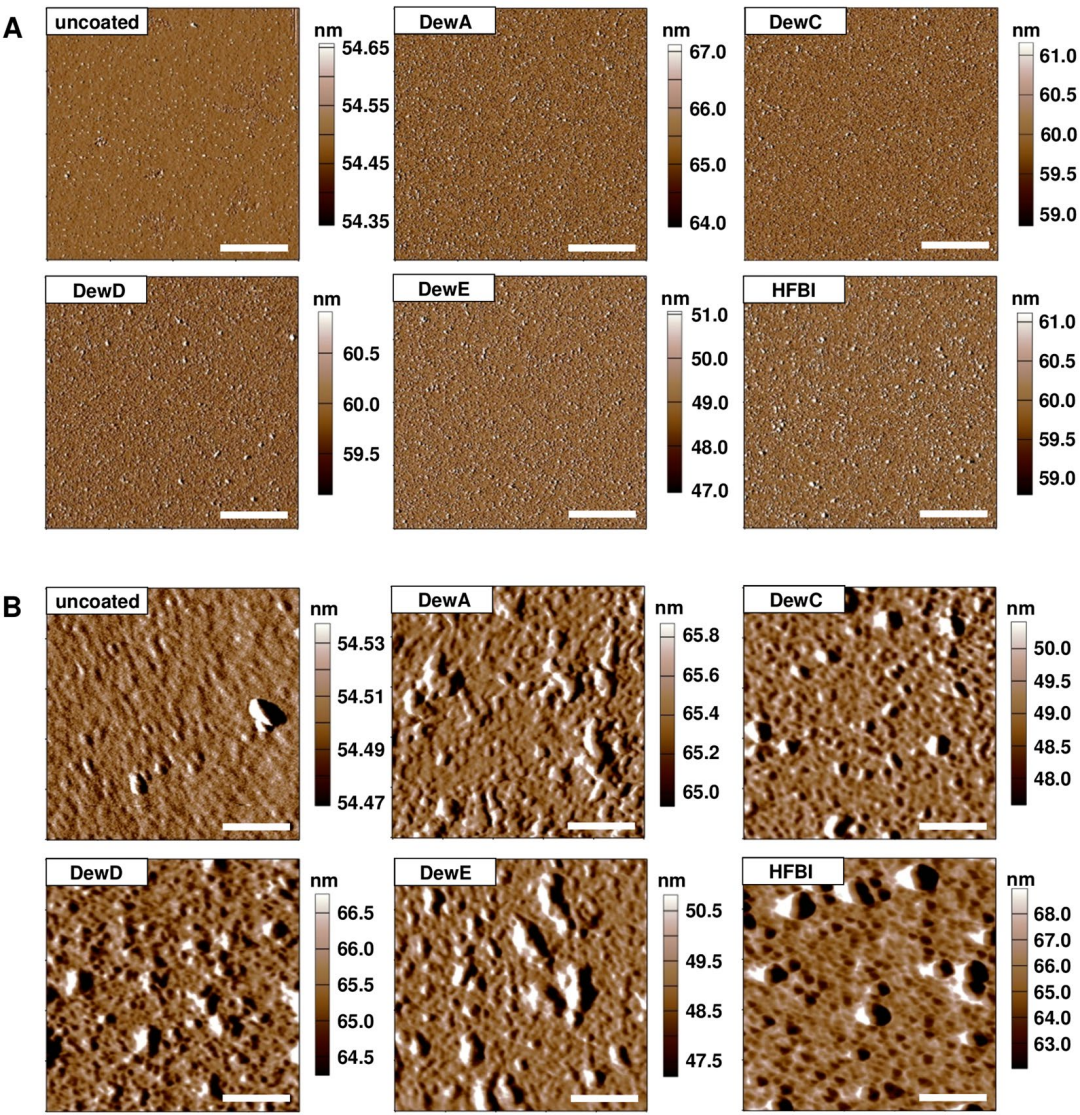
Atomic force microscopy of hydrophobin coated glass surfaces. Depicted are amplitude images of scans of 400 µm^2^ (**A**) and 1 µm^2^ (**B**).

**Figure 4:**
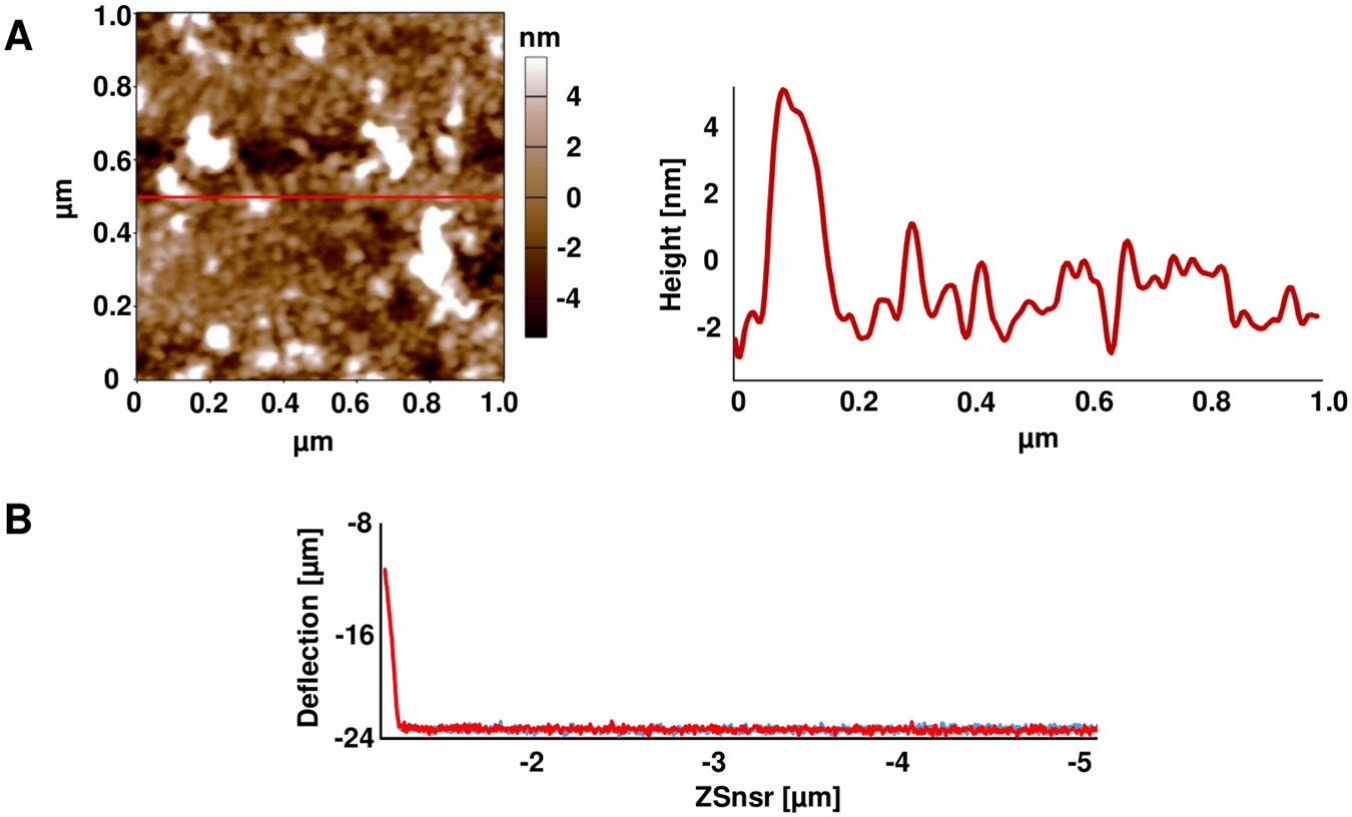
Height and adhesion characterization of the DewA coating. (**A**) AFM height image with height profile. (**B**) Adhesion force measurement of DewA coated glass. Plotted is the displacement between the cantilever and the resting position of the sample surface on the X axis against the deflection of the cantilever on the Y axis. Depicted in red is the convergence, in blue the divergence of the cantilever and the surface.

To analyse the structure of the hydrophobin layer on glass surface, atomic force microscopy (AFM) was performed. Amplitude images with 20 µm side length resembled the ones generated by fluorescence microscopy. A uniformly patterned, 3D-structured protein layer was observed for all five hydrophobin coatings on scans of 400 µm side length and was missing on untreated glass (Fig. 3A). A more detailed view of the surface was generated with scans of 1 µm side length and a slightly higher cantilever resonance frequency (Fig. 3B). Small globular structures of 10 to 20 nm were observed underlying bigger aggregates with an average diameter of 100 nm. Rodlet structures, typical for hydrophobin layers on for example native conidiospore surface^4^, were not observed for the four tested *A*. *nidulans* hydrophobins, or for HFBI from *T*. *reseii*. The height profile of the DewA coating revealed a maximal aggregate thickness of 8 nm (Fig. 4A). This was comparable to the results obtained for DewC, DewD, HFBI, but for DewE a height up to 20 nm could be observed (see Supplementary Fig. S3), which corresponds to the results obtained with immunofluorescence microscopy. Additionally, adhesion force measurements were carried out to check, if the coating of glass with hydrophobins leads to a hard or soft sticky surface. The displacement of the cantilever when pulled back from the DewA-coated surface showed no deflection from the initial trajectory leading to the surface, showing that the modified surfaces had no sticky characteristics (Fig. 4B). Similar results were achieved with other hydrophobins (see Supplementary Fig. S3).

### Determination of the hydrophobic effect of hydrophobin coatings

**Figure 5:**
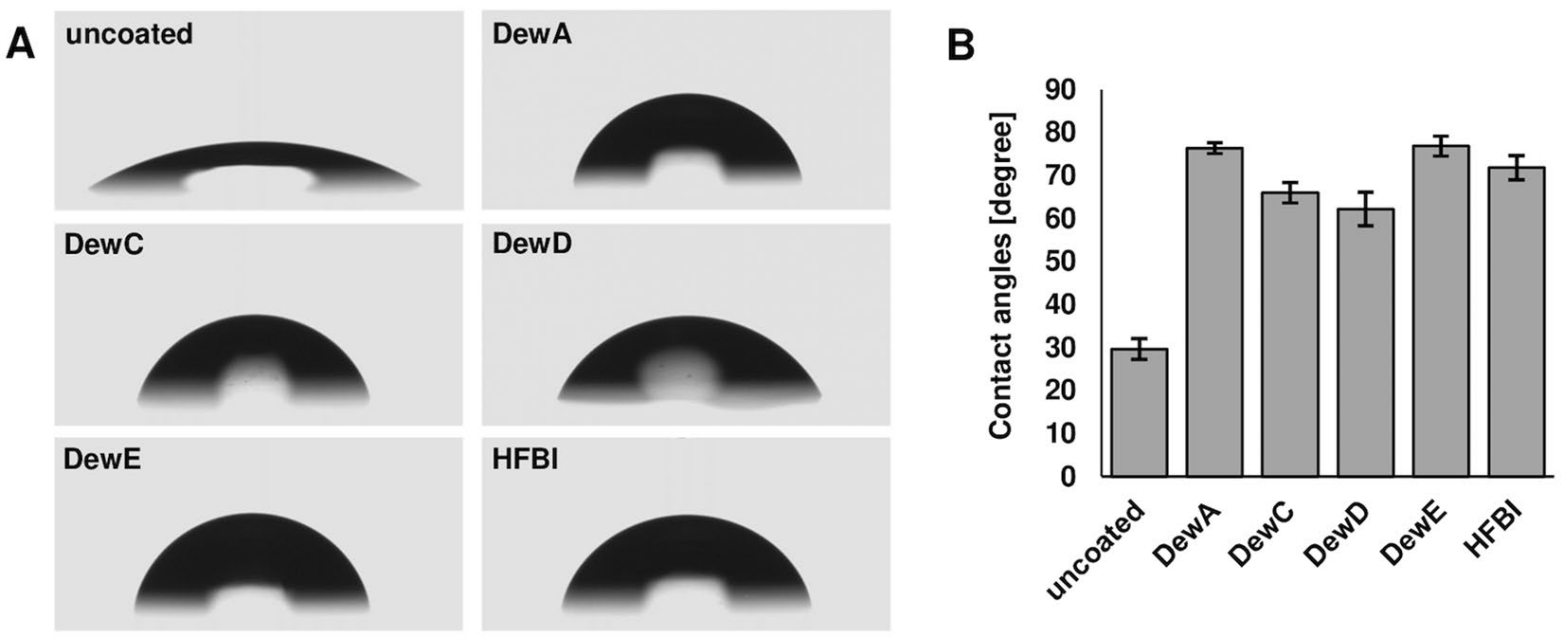
Water contact angle measurements of hydrophobin coated glass slides. (**A**) Pictures of water droplets on bare and hydrophobin coated glass. (**B**) Contact angle measurements of hydrophobin coated glass slides. Data shown as mean ± standard deviation with n = 20.

The static water contact angles of a water droplet on hydrophobin-coated glass slides were measured to analyse the effect of hydrophobins on the hydrophobicity of the glass surface (Fig. 5A). Coatings with class I hydrophobins DewA and DewE generated the most hydrophobic surfaces with measured contact angles of 76.3 ± 0.6° and 76.8 ± 1.2° respectively, compared to the untreated glass with 29.6 ± 2.4° (Fig. 5B). DewC and DewD coated glass showed slightly less hydrophobic effect with 66.0 ± 1.2° and 62.2 ± 1.9° respectively. The hydrophobic effect of the Class II hydrophobin HFBI with a mean contact angle of 71.8 ± 1.4° lied in the middle. Generally, all hydrophobin coatings showed a strong hydrophobic effect on glass.

### Stability of the hydrophobin coatings

**Figure 6:**
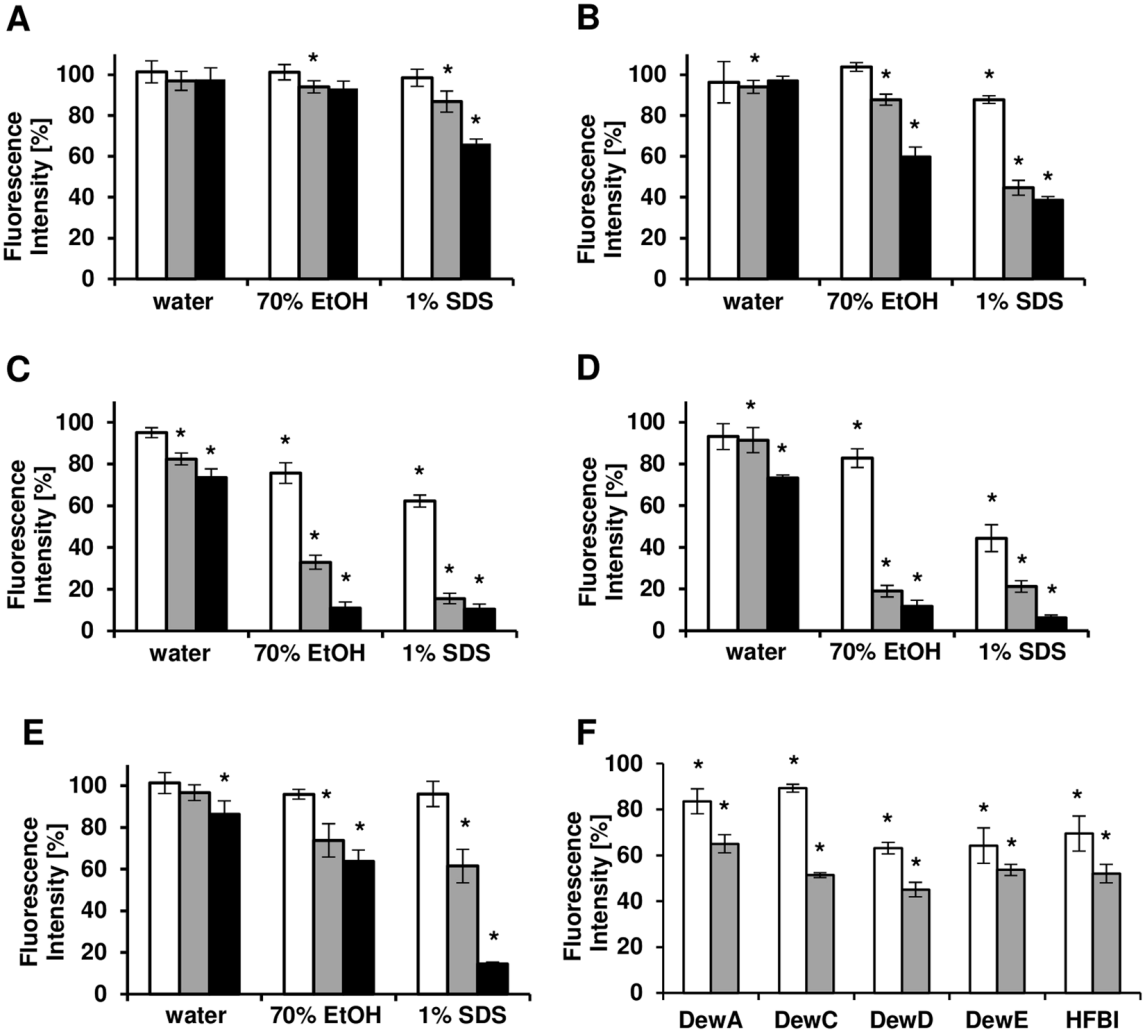
Stability assay of hydrophobin coatings. Diagrams showing the fluorescence intensity of the remaining DewA (**A**), DewC (**B**), DewD (**C**), DewE (**D**) and HFBI (**E**) coating after 1 day (white), 3 days (grey) and 7 days (black) in water, 70% EtOH or 1% SDS. (**F**) Diagram showing the fluorescence intensity of the remaining coating after illumination with UV-C (white) and heating at 80 °C (grey). Data shown as mean ± standard deviation with n = 18 (*p < 0,01).

Alongside the ability of hydrophobins to form uniform layers and modify the characteristics of coated surfaces, the stability of such modification plays an essential role in technical applications of these proteins. Therefore, we have tested the resistance of the coatings on glass towards temperature, UV light, 70% ethanol, 1% SDS detergent and in deionized water. To determine how much protein was left on the surface after the specific treatments, immunofluorescence detection of hydrophobin coatings treated with different solutions was used. For the protein wash-out treatments half of the glass slide wells were not submerged and therefore set as an untreated control with the measured fluorescence intensity defined as 100% for that specific glass slide only (see Supplementary Fig. S4). Three regions of interest on 6 different images were analysed for each condition and time point resulting in a total n = 18. DewA coatings were the most stable towards water, ethanol and SDS treatments (Fig. 6A). After 7 days incubation in water, 97.6% of the fluorescence intensity was maintained. 70% ethanol reduced the fluorescence intensity down to 92.3% and 1% SDS to 66.2%. For DewC coating the incubation in water for 7 days resulted in a loss of 2.9% fluorescence (Fig. 6B). After the treatments with ethanol and SDS only 59.2% and 38.6% of the initial fluorescence signal was detected for this protein. DewD and DewE formed the less stable coatings on glass (Fig. 6C,D). After a week in water, only 73.6% of the coating was left in samples with DewD and 73.3% for DewE. The hydrophobins could be removed nearly entirely with ethanol and SDS. The stability of the class II hydrophobin HFBI coating was comparable to that of DewC for water and ethanol (Fig. 6E). SDS has removed 85.6% of the coating, resulting in a remaining fluorescence intensity of 14.4%.

Illumination with UV-C for 20 min led to a reduction of the hydrophobin coating of 16.4% for DewA, 10.9% for DewC, 36.9% for DewD and 35.8% for DewE (Fig. 6F). HFBI coating was reduced to 69.5%. After the incubation for 2 hours at 80 °C (dry heat), DewA coating was reduced to 65.0%. DewD showed the least stable coating towards heat and only 45.0% of the initial coating was left after baking. DewC, DewE and HFBI showed similar stability towards heat with remaining coatings of 51.4%, 53.6% and 52.1%.

Clearly, though it was previously shown that class I hydrophobins in contrast to class II form highly stable layers on solid surfaces, the hydrophobin affiliation with a certain class doesn’t necessarily determine the stability of the hydrophobin-formed layer. However, the class I hydrophobin DewA showed the highest resistance towards all treatments, as expected.

### Emulsion stabilization by soluble hydrophobins

**Figure 7:**
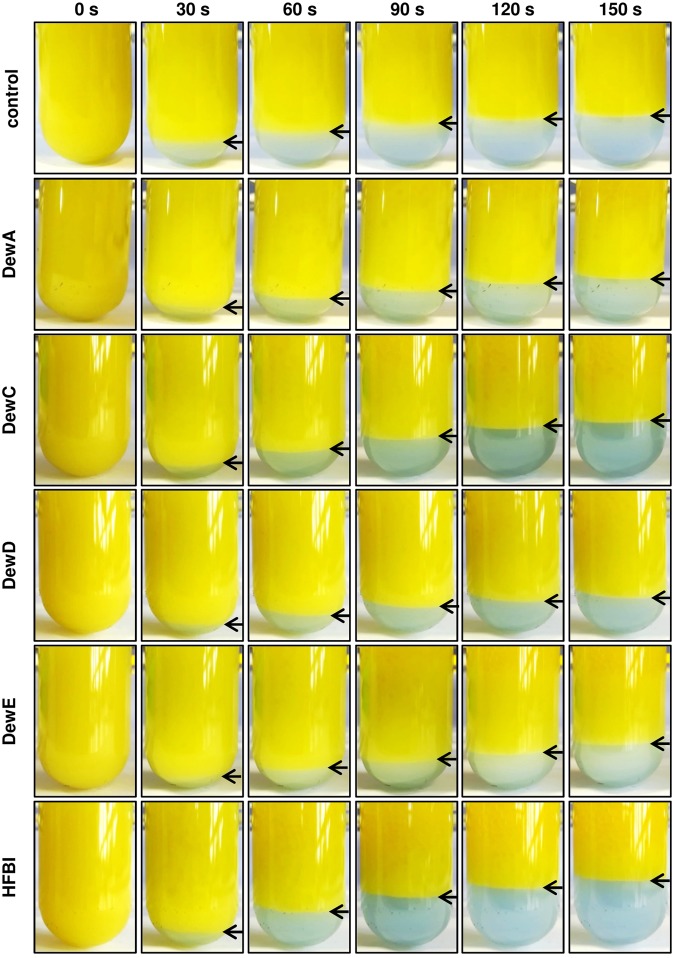
Emulsion stabilization assay. Hydrophobins were dissolved to a final concentration of 200 µg/ml in ddH_2_O and vortexed with the same volume canola oil to generate an homogenous emulsion. Final hydrophobin concentration in the mixture 100 µg/ml. Phase separation was documented by video recording.

Several tested hydrophobins, especially DewD and DewE, formed relatively unstable coating on glass, as demonstrated in the Fig. 6. The possibility to use these proteins in soluble form for emulsion stabilisation was tested on the oil:water mixture. Already 100 µg/ml concentrations of the hydrophobins DewA, DewE and especially DewD have shown a stabilizing effect on the emulsion, compared to the sample without hydrophobin (Fig. 7). However, DewC and HFBI showed no such effect in the given concentration. With the increasing hydrophobin concentration the emulsifying effect was increased for all tested proteins (see Supplementary Fig. S5).

## Discussion

The mechanism of the hydrophobin self-assembly into monolayers on hydrophilic:hydrophobic interfaces has been addressed in various studies in the last years. Both structural characteristics of the proteins and the nature of interface have been shown to influence the layer organization and the formation of rodlets^10,31,32^. Though most studies analysed and compared single or few representatives of class I or II hydrophobins, filamentous fungi often contain several hydrophobins that belong to a single class^33^. It has also been shown that these hydrophobins are often expressed during different stages of organism development, have different localization or exhibit different functions^4,28,34^.

Our results show that hydrophobins from a single organism that belong to the same class can exhibit various surface binding characteristics. The tested hydrophobins from *A*. *nidulans* DewA, DewC, DewD, DewE and HFBI from *T*. *reesei* were all efficient in forming glass surface coatings, thereby increasing the hydrophobicity of glass. Mostly, the hydrophobins formed a uniform layer, with the exception for DewE, which formed protein aggregates, visible both via immunofluorescence and atomic force microscopy. The analysis of the coating resistance towards ethanol, detergent, temperature and UV revealed major differences in the hydrophobin layer characteristics. Only the DewA protein layers showed the class I typical high resistance towards water, ethanol, detergent and temperature treatments. Also, as expected, the HFBI protein, which is a class II hydrophobin, has formed less stable layers on the glass surface. Other hydrophobins from *A*. *nidulans* demonstrated lower resistance towards mentioned treatments than expected. The DewE protein showed most distinctions in both layer formation and stability compared to other tested proteins. It not only formed larger protein aggregates on surface, but was also almost completely removed by both ethanol and SDS treatments that interfere with the hydrophobic interactions between the hydrophobin molecules. This sensitivity could be explained by the nontypical structure of the DewE protein compared to other class I hydrophobins^4^. Though the DewE hydrophathy pattern was previously identified as similar to class I hydrophobins, two hydrophobic unstructured loops that are conserved in typical class I hydrophobins DewA, RodA and DewB from *A*. *nidulans* are shifted in the DewE protein^4^. Another hydrophobin that showed low resistance towards treatments with ethanol and SDS, DewD, has even more distinct hydrophobicity pattern in comparison to other hydrophobins from *A*. *nidulans* and could not be assigned to any class^4^. Both protein layers also showed higher sensitivity towards UV-C and temperature treatments than DewA. On the other hand they showed the best emulsion stabilization effect in oil:water emulsion. The DewC protein, though assigned to class I hydrophobins based on its secondary structure, showed coating stability characteristic close to the HFBI protein.

Recently, the classification into two classes has more and more been questioned and intermediate, unknown or third class of hydrophobins has been proposed, based on the analysis of protein sequences and hydrophobicity profiles^7,8^. Our results underline these analyses with experimental data on coating and emulsifying properties, showing how the structural complexity of these proteins is reflected in the physical characteristics of the hydrophobin constructs. And though some typical class I or II hydrophobins with classical surface coating characteristics can be found in different fungi, many of these proteins, like for example DewC, DewD and DewE from *A*. *nidulans*, elude the typical classification. These proteins exhibit unique structural and functional features. Through further characterization of these untypical hydrophobins, their structure and behavior on different hydrophobic:hydrophilic interfaces, the relationship between their structural characteristics and certain biochemical features and possibly the mechanisms of their action in fungi could be clarified. Also, whereas classical class I hydrophobins like DewA are suitable for functionalization of solid surfaces, the intermediate or unknown class hydrophobins, like DewD and DewE, show potential as foam or emulsion stabilizers in the food industry, as demonstrated by this study.

## Methods

### Strains and growth conditions

The *A*. *nidulans* strain GR5 (*pyrG89; wA3; pyroA4; veA1*)^35^ was cultivated for two days in supplemented liquid minimal medium (MM)^36^ before RNA extraction. *Escherichia coli* strains TOP10 (Invitrogen, Karlsruhe, Germany) and SHuffle ® T7 (New England Biolabs, Frankfurt am Main, Germany) were used for molecular biology techniques and protein expression respectively. Standard *E*. *coli* cultivation was carried out in lysogeny broth (1% trypton, 0.5% yeast extract, 0.5% NaCl)^37^, protein expression in EC3 medium: 1.5% trypton, 1.5% yeast extract, 3% glycerol, 0.2% KH_2_PO_4_, 0.5% (NH_4_)_2_SO_4_, 0.1% MgSO_4_ x 7 H_2_O, 0.01% CaCl_2_ x 2 H_2_O, 0,1% SL4 trace elements^15^.

### Plasmid construction

For the amplification of the *A*. *nidulans* hydrophobin genes *dewA*, *dewC*, *dewD* and *dewE* from cDNA, the mycelium from a two days old *A*. *nidulans* GR5 culture was filtered through miracloth (Merck KGaA, Darmstadt, Germany), grinded in liquid nitrogen and RNA was isolated with the E.Z.N.A. Fungal RNA Kit (Omega Biotek, Norcross, USA). After DNA digestion with the TURBO DNA-free^TM^ Kit (Thermo Fisher Scientific, Waltham, USA), cDNA was synthesized with the SuperScript ™ IV First Strand Synthesis System (Thermo Fisher Scientific, Waltham, USA). HFBI coding gene was synthesized *in vitro* (Integrated DNA Technologies, Leuven, Belgium), sub cloned in pJET1.2 vector (Thermo Fisher Scientific, Waltham, USA) and amplified by PCR. PCR fragments were cloned into pET28a vector (Novagen, Darmstadt, Germany), carrying a C-terminal His-tag, under the IPTG-inducible (isopropyl β-D-1-thiogalactopyranoside) T7 promotor using restriction enzymes SphI and HindIII. In a second step, the leader sequence from *Erwinia carotovora* pectate lyase B (pelB)^29^ was generated by primer dimerization forming duplex DNA and then cloned N-terminally to the hydrophobin genes using NcoI and SphI restriction sites. All primers used are listed in Table 1.

**Table 1:** Primers used in this study.

Primer	Sequence (5′-3′)
DewA fw	ATGCATGCGCTTCATCGTCTC
DewA rev	ATAAGCTTA*GTGATGGTGATGGTGATG*AGATCCCTCAGCCTTGGTACCAG
DewC fw	ATGCATGCAATTCACAATCGC
DewC rev	ATAAGCTTA*GTGATGGTGATGGTGATG*AGATCCGAGAACCTGGACAGGAAC
DewD fw	ATGCATGCATCTTTCCACCTCC
DewD rev	ATAAGCTTA*GTGATGGTGATGGTGATG*AGATCCCTTGTCAACGCCATCAC
DewE fw	ATGCATGCTTATGAAGGTCGCCACTGC
DewE rev	ATAAGCTTA*GTGATGGTGATGGTGATG*AGATCCGTGGCCGTGCTCCAG
HFBI fw	ATGCATGCAGCCGTTAGAAGATC
HFBI rev	ATAAGCTTA*GTGATGGTGATGGTGATG*AGATCCTGCGCCCACGGC
pelB fw	AACCATGGATGAAATCGTTCATCGCGCCGATCGCTGCGGGCCTGCTGCTGGCCCTGTCTCAGCCGCTGCTGGCTGGCATGCAA
pelB rev	TTGCATGCCAGCCAGCAGCGGCTGAGACAGGGCCAGCAGCAGGCCCGCAGCGATCGGCGCGATGAACGATTTCATCCATGGTT

The underlined sequences represent the restriction sites for cloning. The italicized sequences represent the 6xHis tag.

### Protein expression and purification

Protein expression from recombinant *A*. *nidulans* hydrophobins DewA, DewC, DewD and DewE and HFBI from *T*. *reseii* was carried out in SHuffle ® T7 Competent *E*. *coli* cells. 500 ml EC3 medium supplemented with 50 µg/ml kanamycin were inoculated to an OD_600_ = 0.05 with an overnight culture and incubated at 37 °C and 180 rpm. Upon reaching an OD_600_ = 0.6 protein expression was induced by addition of isopropyl-*β*-D-thiogalactopyranosid (IPTG) to a final concentration of 0.5 mM. Cells were harvested the next day and purification from inclusion bodies was performed at alkaline pH without the addition of urea as described previously^15^. Solubilized hydrophobins were stored at −20 °C.

### Surface coating

The surface coating procedure was adapted from Rieder *et al*.^25^, with following modifications. Prior to coating glass slides were washed for 30 min in 100% isopropanol, rinsed in deionized H_2_O and dried under air flow. Aqueous solutions with 50, 100, 200 and 500 µg/ml hydrophobin in coating buffer (50 mM Tris pH 8.0, 1 mM CaCl_2_) were applied on the glass slides with the hydrophobin of choice and incubated at 60 °C for 2 hours in a humidity chamber to avoid complete evaporation of the coating solution. After incubation the protein solution was removed and the surfaces were subsequently washed three times for 10 min with distilled water and air-dried at room temperature.

### Immunofluorescence microscopy

For specific immunodetection of the hydrophobin layers uncoated and hydrophobin-coated glass slides were blocked for 30 min with 10% milk in TBS (Tris-buffered saline) at room temperature. The primary α-His antibody (Thermo Fisher Scientific, Waltham, USA), diluted 1:2000 in 1% milk in TBS was applied for 1 hour. The glass slide surface was washed four times for 5 minutes in TBS and the secondary Cy3-labelled antibody to mouse IgG (Dianova GmbH, Hamburg, Germany) was applied in 1:5000 dilution in 1% milk in TBS for 1 hour in the dark and washed in TBS and water. Imaging was carried out on an AxioImagerZ.1 with an AxioCam MRm camera and the software Zen Pro 2012 (Carl Zeiss AG, Oberkochen, Germany) with the excitation wavelength of 552 nm and emission wavelength of 565 nm.

### Atomic force microscopy

Atomic force microscopy (AFM) imaging was carried out on a MFP-3D AFM (Asylum Research, Santa Barbara, USA) with the software Igor Pro 6.35A5 (WaveMetrics, Portland, USA). The imaging process was conducted in tapping mode. Silicon cantilevers (BudgetSensors, Sofia, Bulgaria) were operated at a resonance frequency in the range of 65 kHz to75 kHz possessing an average spring constant of 3 N/m.

### Determination of water contact angles

The static water contact angles of uncoated and coated glass surfaces were measured with an OCA20 and the software SCA 202 v3.12.11 (both DataPhysics Instruments GmbH). 4 µl deionized water drops were put on the surfaces by the “hanging drop” method and imaged with a CCD camera with a resolution of 768 × 576 px. An ellipse fit was chosen to approach the droplet form, followed by the determination of the contact angles.

### Coating stability assay

To define the stability of the different hydrophobin coatings, the wells of epoxy-coated 8-well diagnostic slides (Thermo Fisher Scientific, Waltham, USA) were coated with hydrophobin. The slides were submerged half in deionized water, 70% ethanol or 1% SDS (sodium dodecyl sulfate) solution for up to 7 days at room temperature (see Figure S4 for experimental setup).

After 1, 3 and 7 days, slides were rinsed with water and hydrophobin coating was visualized by fluorescence microscopy. For UV stability, coated slides were illuminated for 20 min in an UV-Crosslinker BLX-E254 (Vilber Lourmat, Marne-la-Vallée, France) emitting UV-C with 254 nm. For temperature stability, coated slides were heated to 80 °C for 2 h in a hybridization oven and then analysed by fluorescence microscopy. Fluorescence intensity of taken images was measured with ImageJ. A total of three regions of interest for six images per condition was measured (n = 18). Values given are remaining coating intensity of submerged wells compared to the untreated wells on the same slide. Statistics were carried out using the two-tailed student’s t-test assuming equal variance with an alpha level of 0,01. Data were tested for normality using the Anderson-Darling test.

### Emulsion stability assay

Due to the amphiphilic character of hydrophobins, their use as emulsifying reagents is widely analysed. Here we chose a simple setup to assess the property of the purified hydrophobins to form stable oil-water emulsions. Hydrophobins were dissolved in water dyed with 25 µg/ml Remazol Brilliant Blue R for better visualization of the aqueous phase. 2 ml of canola oil were vortexed with 2 ml hydrophobin solution for 30 s (final hydrophobin concentrations 100 µg/ml and 500 µg/ml) and phase separation was documented by video recording.

### Data availability statement

The datasets generated or analysed during this study are included in this published article (and its Supplementary Information file) or are available from the corresponding author on request.

## Electronic supplementary material

Supplementary Information
