# Supplementary material for: Comparative analysis of surface coating properties of five hydrophobins from Aspergillus nidulans and Trichoderma reseei
Source: Sci Rep. 2018 Aug 13;8:12033. doi: 10.1038/s41598-018-29749-0 (PMC6089913; doi:10.1038/s41598-018-29749-0)
Supplement: Supplementary file 1 — Supplementary Information [file 41598_2018_29749_MOESM1_ESM.docx]

**Supplementary Information**

**Comparative analysis of surface coating properties of five hydrophobins from *Aspergillus nidulans* and *Trichoderma reseei***

Lex Winandy^1^, Felix Hilpert^2^, Oleksandra Schlebusch^1^* and Reinhard Fischer^1^

^1^ Department of Microbiology, Institute for Applied Biosciences, Karlsruhe Institute of Technology (KIT), Karlsruhe, Germany

^2^ Institute of Chemical Process Engineering, Mannheim University of Applied Sciences, Mannheim, Germany

*Corresponding author

Oleksandra Schlebusch

Email: oleksandra.schlebusch@kit.edu


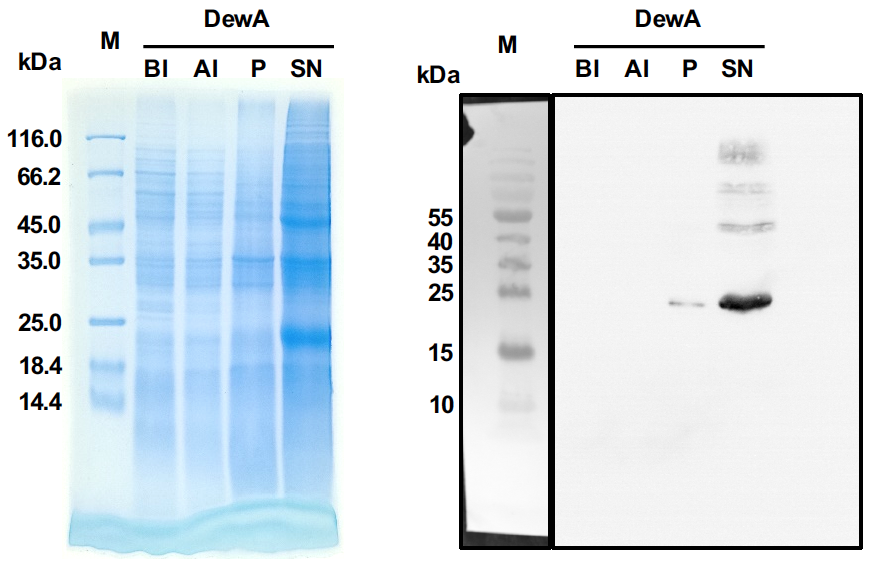


**Supplementary Fig. S1.** **Recombinant hydrophobin purification.**Full scale images of the purified DewA detection by Coomassie stained 15% SDS polyacrylamide gel and immunoblot detection. Immunoblot image is composed of the photograph of a prestained protein ladder and the protein immunoblot picture after 15 sec exposure in the darkness from the same membrane. M - marker, BI - before induction, AI - after induction, P - pellet after inclusion body purification, SN –soluble protein in supernatant after purification.


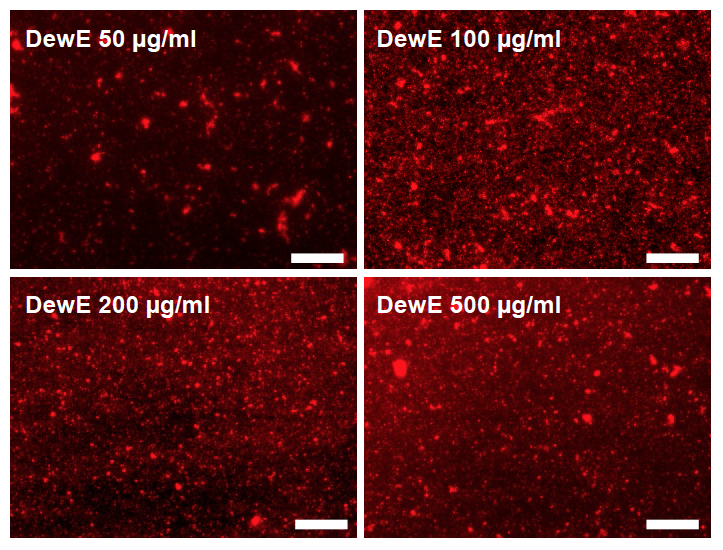


**Supplementary Fig. S2. DewE glass coating.** DewE coated glass slides with different protein concentrations in the coating solution. Scale bar = 20 µm.


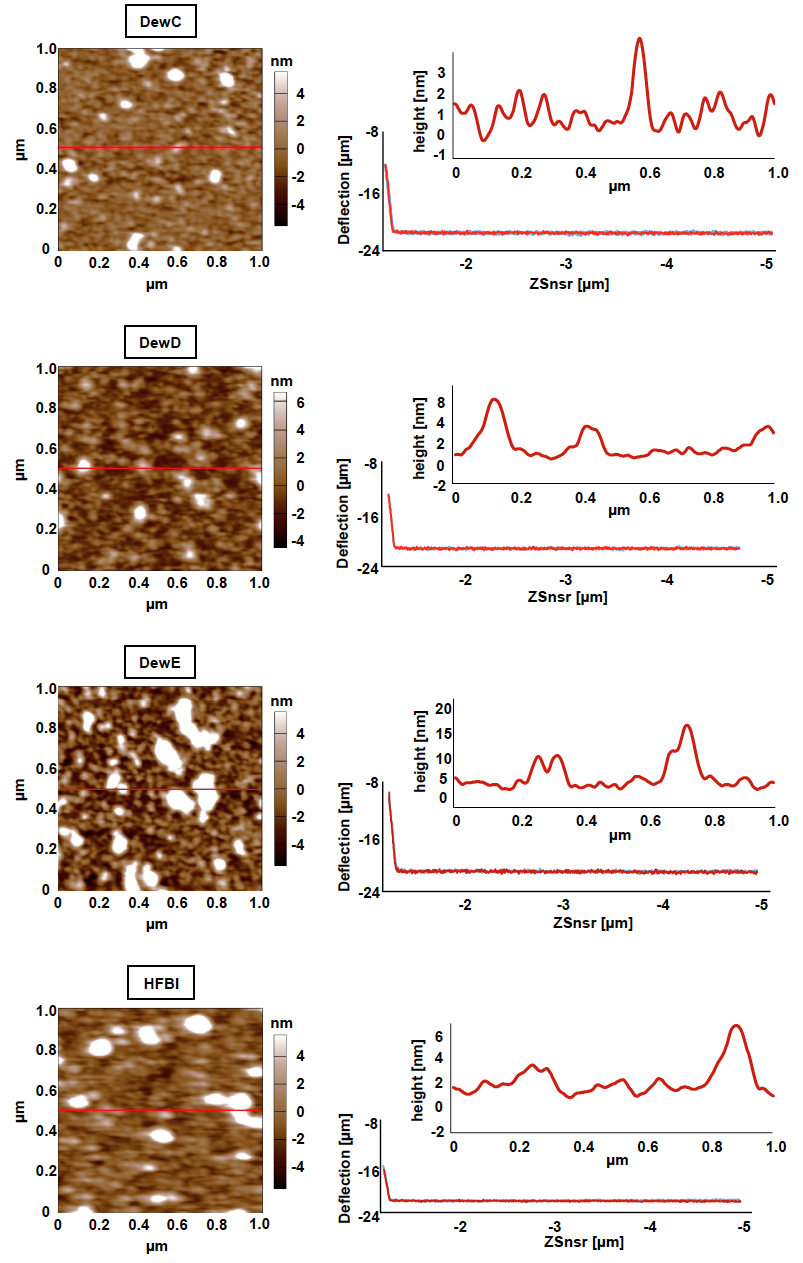


**Supplementary Fig. S3.** **Atomic force analysis of DewC, DewD, DewE and HFBI coated glass.** AFM height images with height profile and adhesion force measurements.

**
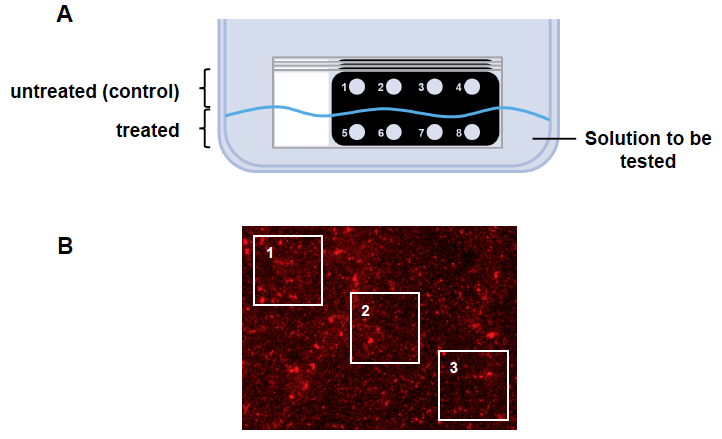
**

**Supplementary Fig. S4.** **Coating stability assay.** A) Scheme of the setup used for the determination of the stability of hydrophobin coatings when treated with water, 70% EtOH or 1% SDS for up to 7 days. With hydrophobin coated masked glass slides are immerged by half in the solution to be tested. B) Figure representing the 3 regions of interest measured for each of the images taken for fluorescence intensity measurements.


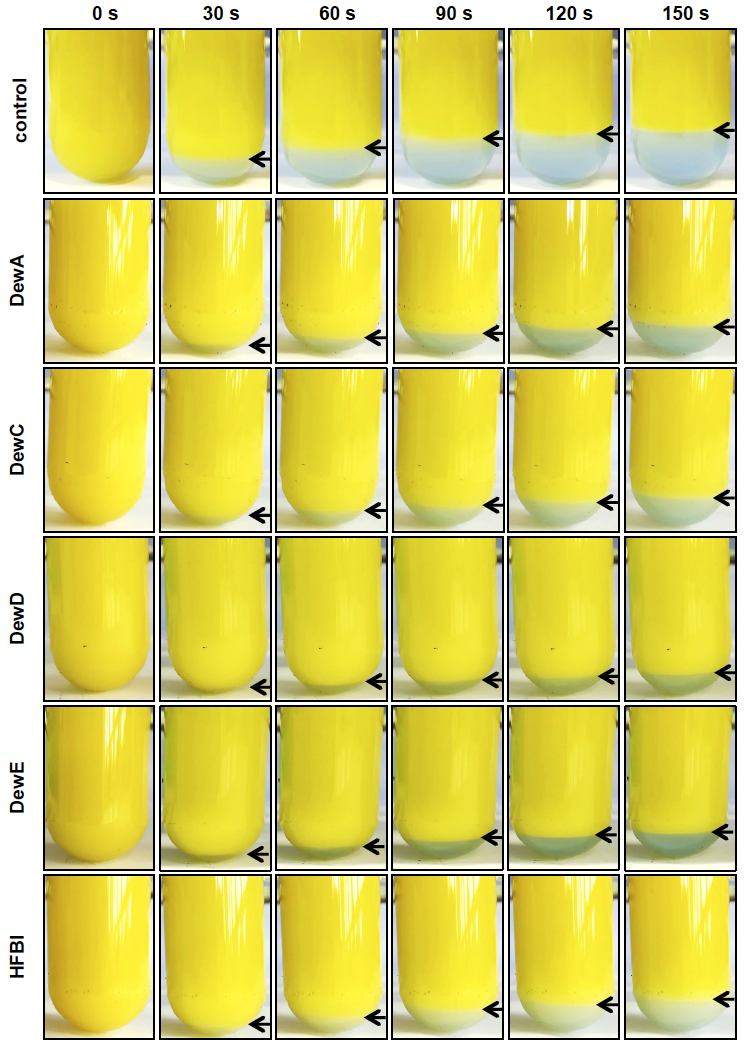


**Supplementary Fig. S5. Emulsion stabilization assay with 500 µg/ml hydrophobins.** Hydrophobins were dissolved to a final concentration of 200 µg/ml in ddH_2_O and vortexed with the same volume canola oil to generate a homogenous emulsion. Final hydrophobin concentration in the mixture 100 µg/ml. Phase separation was documented by video recording.
